# Supplementary material for: A Niclosamide-releasing hot-melt extruded catheter prevents Staphylococcus aureus experimental biomaterial-associated infection
Source: Sci Rep. 2022 Jul 19;12:12329. doi: 10.1038/s41598-022-16107-4 (PMC9296466; doi:10.1038/s41598-022-16107-4)
Supplement: Supplementary file 1 — Supplementary Information. [file 41598_2022_16107_MOESM1_ESM.pdf]

Instrumental Parameter	Value
2-theta range	2-45°
Step size [°2-theta]	0.0167
Time per step [sec]	59.690 sec
Scan Mode	Continuous
Sample Movement	Spinning, 1.0sec rotation time
Wavelength [nm]	Cu $K\alpha_1$ = 1.54060 $K\alpha_2$ = 1.54443
X-ray Mirror	Inc. Beam Cu W/Si focusing MPD, Acceptance Angle 0.8°C, Length 55.3 mm
Incident Divergence Slit<br>Incident Antiscatter Slit<br>Incident Beam Mask<br>Beam Stop	Slit Fixed 1/2°<br>Slit Fixed 1/2°<br>10 mm<br>Beam Stop for Transmission Spinner
Temperature/RH	Room temperature
Soller Slits	0.02 rad on Incident and Diffracted beam
Detector type	X'Celerator (active length 2.122°)
Sample holder	Transmission sample holder. Samples are mounted between Mylar film
Configuration	Transmission
Generator voltage/current	40 KV / 40 mA

Supplementary Table 1. Instrumental parameters. The XRD analyses were run in transmission mode on an X-Ray Diffractometer equipped with an X'Celerator detector using a standard XRD method.

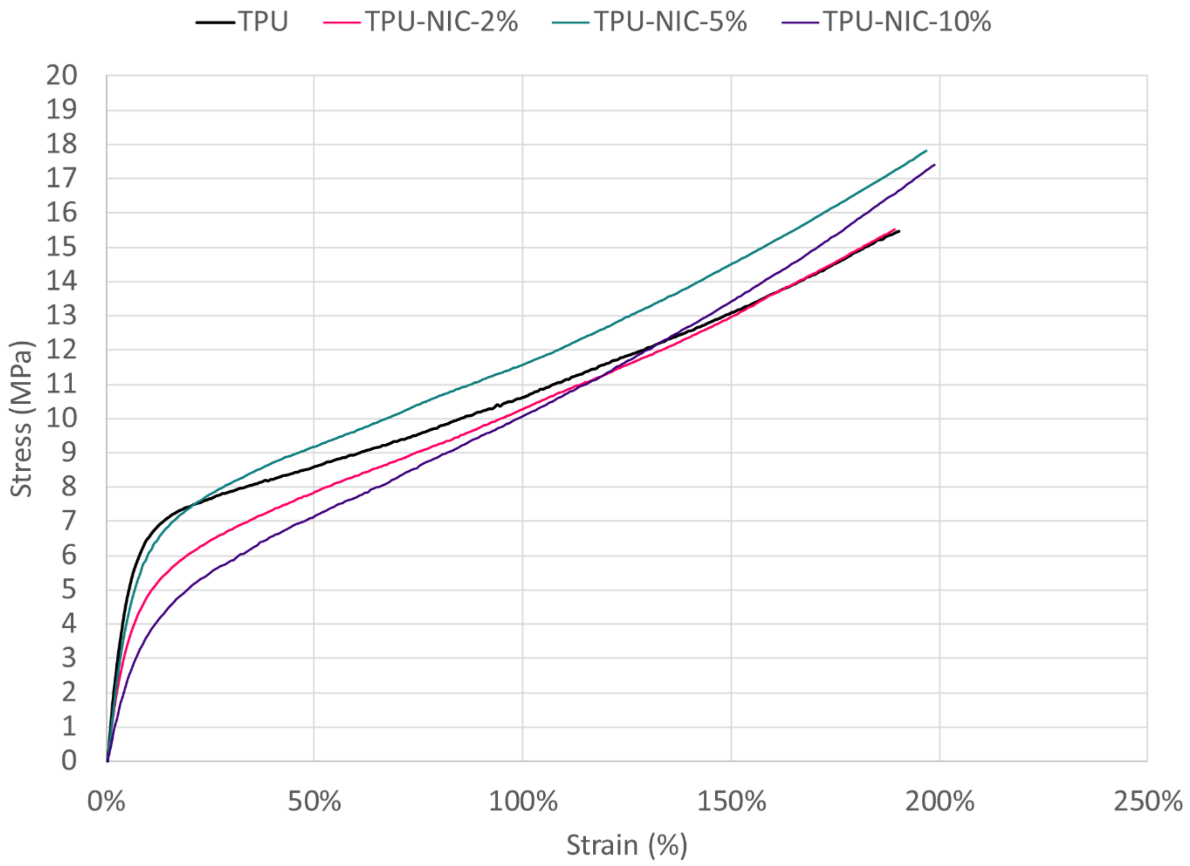

Supplementary figure 1. Representative stress strain curve generated from tensile test of TPU fibers loaded with 0 , 2, 5 and 10 % of niclosamide.

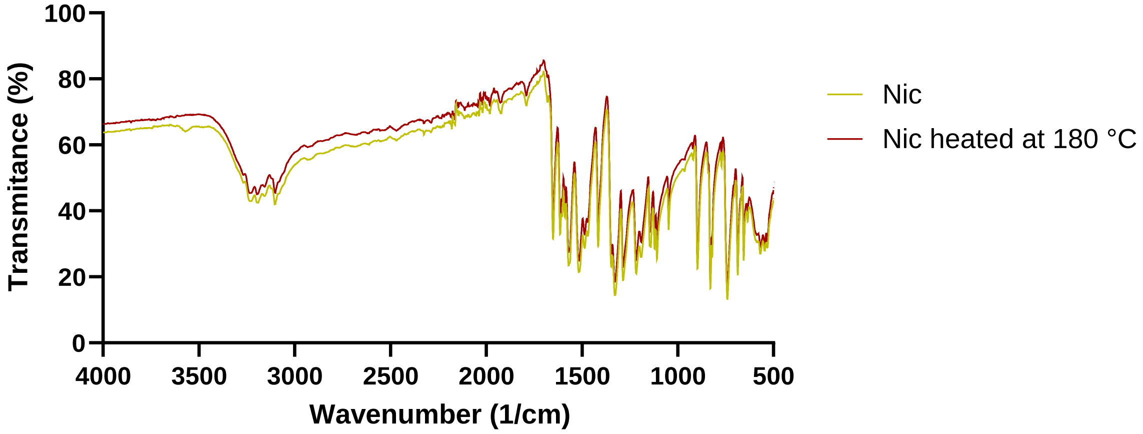

Supplementary figure 2. FTIR spectra of neat Nic (yellow line) and heated for 5 min at 180 °C (red line).
